# A real-world direct cost associated with a 4-year postdiagnosis follow-up in a population-based cohort of patients with melanoma by clinical–pathological characteristics

**DOI:** 10.1097/CMR.0000000000001033

**Published:** 2025-03-28

**Authors:** Alessandra Buja, Massimo Rugge, Carlo Maria Formaro, Giulia Grotto, Claudia Cozzolino, Antonella Stefano, Manuel Zorzi, Antonella Vecchiato, Paolo Del Fiore, Saveria Tropea, Chiara Trevisiol, Carlo Riccardo Rossi, Simone Mocellin

**Affiliations:** a Department of Cardiac, Thoracic, Vascular Sciences, and Public Health, University of Padua; b Veneto Tumor Registry (RTV), Azienda Zero; c Department of Medicine – DIMED, Pathology and Cytopathology Unit, University of Padua; d Soft-Tissue, Peritoneum and Melanoma Surgical Oncology Unit, Veneto Institute of Oncology IOV-IRCCS; e Department of Surgery, Oncology and Gastroenterology – DISCOG, University of Padua, Padua, Italy

**Keywords:** healthcare costs, health expenditures, melanoma

## Abstract

In times of limited resources, data on the costs of disease should be one of the primary factors assisting policymakers in attaining the best value for money. This study aimed to analyze the real-world direct costs associated with a 4-year postdiagnosis follow-up of a population-based cohort of patients with cutaneous melanoma stratified by sociodemographic and clinical characteristics. The cost analysis was conducted from the perspective of the health system. Data on visits to outpatient clinics, specialist services, drug prescriptions, hospital or hospice admissions, and treatments at the emergency department were obtained from the regional administrative subject-level databases (see below). The cost of any diagnostic or therapeutic (surgical or otherwise) interventions was based on the reimbursement rates established by the Veneto Regional Authority. This study revealed that direct healthcare costs for patients with melanoma are associated with sociodemographic characteristics, that is, male gender and older age, and anatomopathological factors such as tumor-node-metastasis (TNM) stage, mitotic count, and growth pattern, with the highest costs occurring in vertical growth melanoma. Given the rising incidence of melanoma, the analysis of real-world direct costs for a population-based cohort of patients is essential for informing decision-makers on how to better allocate healthcare resources.

## Introduction

With around 350 000 new cases in the world in 2020, melanoma of the skin is estimated to account for 1.7% of all new malignant tumors [1]. In 2020, the age-standardized global incidence was 12.2 per 100 000 people. The worldwide incidence of melanoma has continued to increase in developed, predominantly fair-skinned countries over the past decades [1]. Incidence in the US rose by more than 320%, from 7.9 per 100 000 in 1975 to 25.3 per 100 000 in 2018 [1], making melanoma the fifth most common cancer diagnosis and representing 5.0% of all new cancer cases [2]. In other words, melanoma has not only become a major health problem but also an economic concern.

In times of limited resources, data on the costs of disease should be one of the primary factors assisting policymakers in attaining the best value for money and ensuring an efficient allocation of public resources across different services and care pathways [3]. Many studies have estimated the monetary burden of melanoma on the basis of clinical guidelines [4–7]. A limitation of such studies is that they do not necessarily reflect what actually occurs in real-world healthcare because physicians do not always adhere to standardized protocols and patients are not always compliant. Analyzing real-world data can provide important insights and enable the cancer burden to be analyzed.

In the case of melanoma, there is still a paucity of studies describing resource utilization and costs in routine clinical practice based on real-world data [8]. A previous study found that the annual cost of patient care ranges from 149 euros (€) for patients with in-situ melanoma to €66 950 for some patients with stage IV disease [4]. Meanwhile, an Australian study estimated that the annual cost of a patient with melanoma ranges from $647 per patient with stage I to $119 070 per patient with stage IV disease [9]. To the best of our knowledge, very few research studies have compared the costs of the patient with melanoma treatment based on their sociodemographic and clinical characteristics.

This study aimed to analyze the real-world direct costs associated with a 4-year postdiagnosis follow-up for a population-based cutaneous melanoma (CMM) cohort of patients stratified by sociodemographic and clinical characteristics.

## Methods

### Context

The Italian public healthcare system is managed regionally and provides universal coverage that is mostly free at the point of delivery. It is primarily funded by general taxation [10]. Its policies are grounded on the fundamental values of universality, free access, freedom of choice, pluralism in provision, and equity.

Veneto is an Italian region located in the north-eastern area of the country with a population of 4.9 million residents and a mean age of 54.4 years. According to the most recent socioeconomic indicators, Veneto has, in 2022, a per capita gross domestic product of €37 238.2 [11] and an unemployment rate of 13.4% [12]. In 2018, 36.9% of women and 27% of men aged between 30 and 34 were university graduates [13–15]. In 2015, the Veneto Oncology Network (ROV) detailed the complete procedures for the clinical management of patients with CMM in a document based on the current national and international literature. This document defines standardized clinical care pathways from diagnosis to end-of-life care as well as a set of indicators to assess the quality of the care process, evaluating the consistency between recommendations and real-world clinical practice [16].

### Clinical and cost data

This population-based cohort study includes all incident cases of cutaneous malignant melanoma reported in 2015 and 2017 and recorded by the Veneto Cancer Registry (*Registro Tumori Veneto* or RTV), a high-resolution regional cancer registry covering the region’s entire population.

RTV registration procedures are based on various sources of information, including pathological reports, medical records, death certificates, and administrative records of health services. For the present investigation, the following categorized variables were retrieved from the high-resolution registry: age (<18, 18–64, and ≥65 years), sex (male and female), American Joint Committee on Cancer (AJCC) eighth edition TNM stage at diagnosis, histological subtypes of CMM (superficial spread, nodular, lentigo maligna/spitzoid melanoma, and other), mitotic count per mm^2^ (0, 1–6, and >6) and type of growth (vertical or radial) [14,16]. The cost analysis was conducted from the perspective of the Italian health system. Data on visits to outpatient clinics, specialist services, drug prescriptions, hospital or hospice admissions, and treatments at the emergency department were obtained from the regional administrative subject-level databases (see below). The cost of any diagnostic or therapeutic (surgical or otherwise) interventions was based on the reimbursement rates established by the Veneto Regional Authority. The following sources were consulted for the cost assessment:

1. The outpatient database, which contains information on all medical procedures (specialist visits, laboratory and radiological tests, radiotherapy and chemotherapy sessions, etc.) delivered at National Health Service–funded outpatient facilities and valued at the rate stated in the Tariff Nomenclature for Outpatient Services, a detailed formulary of outpatient medical procedures [17].
2. The hospital admissions database, which includes the diagnosis-related group for each admission, is valued at the rate indicated in the Tariff Nomenclature for Inpatient Services, a formulary encompassing all hospital activities, including day hospital admissions [18].
3. The regional database of in-hospital drug consumption, which records the costs of all medical therapies (including their dosages).
4. The emergency department admissions database, which records the cost of each admission as the sum of all medical procedures undertaken.
5. The hospice database, which records the admission length of stay.

Note that outpatient drugs and medical devices were excluded from the cost analysis because they were difficult to classify as melanoma-related or unrelated expenses. Each patient’s administrative data were associated with an anonymous, unique identifier code. All costs are reported in €.

### Statistical analysis

Absolute frequencies and percentages were used for the descriptive statistics of categorical variables, while continuous numerical variables were expressed by means, medians, and minimum–maximum intervals.

The total number of mortality events observed following a diagnosis of CMM was obtained for the entire sample by linking the high-resolution register with the mortality register.

Cost data were utilized to calculate the annual direct melanoma-related costs per patient from the 2 years preceding the melanoma diagnosis to the 4 years that followed, adjusted per person-years.

Costs were summarized as survival-weighted means and medians (calculated by withering the cost for patients’ survival times); 95% confidence intervals (CIs) for the mean and minimum–maximum intervals were also provided.

Data preparation, analyses, and visualizations were conducted using R 4.2.2 (R Core Team, Vienna, Austria) [19] and Python 3.8.18 (Python Software Foundation, Wilmington, Delaware, USA) [20].

## Results

This study comprised a total of 2647 cutaneous malignant melanoma incident cases: 1279 cases were diagnosed in 2015 (48.3%) and 1368 in 2017 (51.7%). The mean age at diagnosis was 59.7 (SD ± 16.2) years and 53.0% of patients were male. The characteristics of the two cohorts are shown in Table 1. Less than 15% of patients presented an advanced TNM stage at diagnosis (9.9% in stage III and 3.5% in stage IV).

**Table 1:** Patients characteristics by year of diagnosis.

Patients’ characteristics	Diagnosis year		Overall cohort
	2015 (*N* = 1279)	2017 (*N* = 1368)	(*N* = 2647)
Age			
Median (IQR)	58.00 (46.00–72.00)	61.00 (48.00–74.00)	60.00 (47.00–73.00)
Mean (SD)	58.67 (16.25)	60.73 (16.04)	59.74 (16.17)
Sex, *n* (%)			
Female	601 (47.0%)	642 (46.9%)	1243 (47.0%)
Male	678 (53.0%)	726 (53.1%)	1404 (53.0%)
Stage, *n* (%)			
I	922 (72.1%)	905 (66.2%)	1827 (69.0%)
II	161 (12.6%)	218 (15.9%)	379 (14.3%)
III	120 (9.4%)	141 (10.3%)	261 (9.9%)
IV	26 (2.0%)	67 (4.9%)	93 (3.5%)
Missing	50 (3.9%)	37 (2.7%)	87 (3.3%)
Melanoma histological subtypes, *n* (%)			
Lentigo maligna or spitzoid	56 (4.4%)	62 (4.5%)	118 (4.5%)
Nodular	159 (12.4%)	206 (15.1%)	365 (13.8%)
Other	136 (10.6%)	152 (11.1%)	288 (10.9%)
Superficial spreading	928 (72.6%)	948 (69.3%)	1876 (70.9%)
Grow type, *n* (%)			
Radial	285 (22.3%)	270 (19.7%)	555 (21.0%)
Vertical	701 (54.8%)	804 (58.8%)	1505 (56.9%)
Missing	293 (22.9%)	294 (21.5%)	587 (22.2%)
Mitosis per mm^2^, *n* (%)			
0	781 (61.1%)	737 (53.9%)	1518 (57.3%)
1–6	385 (30.1%)	475 (34.7%)	860 (32.5%)
>6	113 (8.8%)	156 (11.4%)	269 (10.2%)

IQR, interquartile range.

Only 13.7% of the cohort died at 4 years after diagnosis. However, significant differences were observed in survival stratification according to TNM stage: while more than 95% of stage I patients survived, only 28% of stage IV patients survived 4 years, while 26.91% of stage II patients died 4 years after diagnosis and 36.78% of stage III patients.

Considering the time trend in the estimated real-world survival-weighted mean cost per patient (Table 2), the highest costs were incurred during the first year following a CMM diagnosis (€2963, 95% CI: 2667–3258), followed by a gradual decrease (€1885, 95% CI: 1583–2187 in the fourth year).

**Table 2:** Cutaneous melanoma-specific survival-weighted mean cost per patient (in euros) by time from diagnosis.

Time from diagnosis	Mean (95% CI)	Median (IQR)
2 Years pre	144 (129–160)	20 (122)
1 Year pre	425 (391–459)	180 (345)
1 Year	2963 (2667–3258)	1278 (2192)
2 Years	2308 (1971–2646)	472 (774)
3 Years	2038 (1710–2366)	407 (616)
4 Years	1885 (1583–2187)	406 (661)

CI, confidence interval; IQR, interquartile range.

**Fig. 1:**
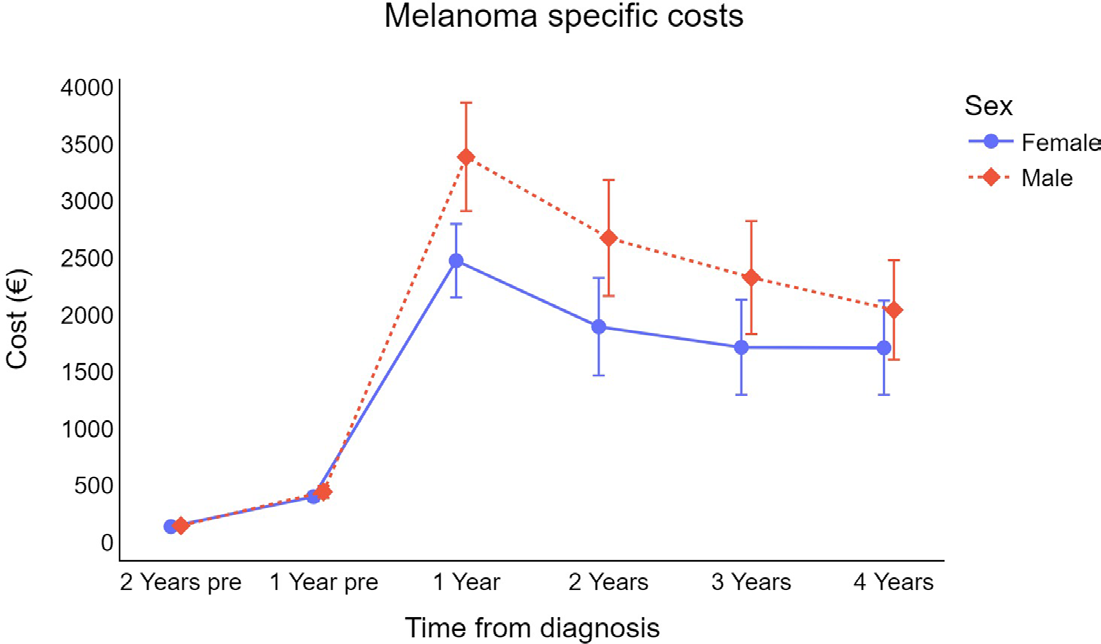
Trend for cutaneous melanoma survival-weighted mean cost per patient (in €) by sex (error bars represent 95% CI for the mean). €, euros; CI, confidence interval.

**Fig. 2:**
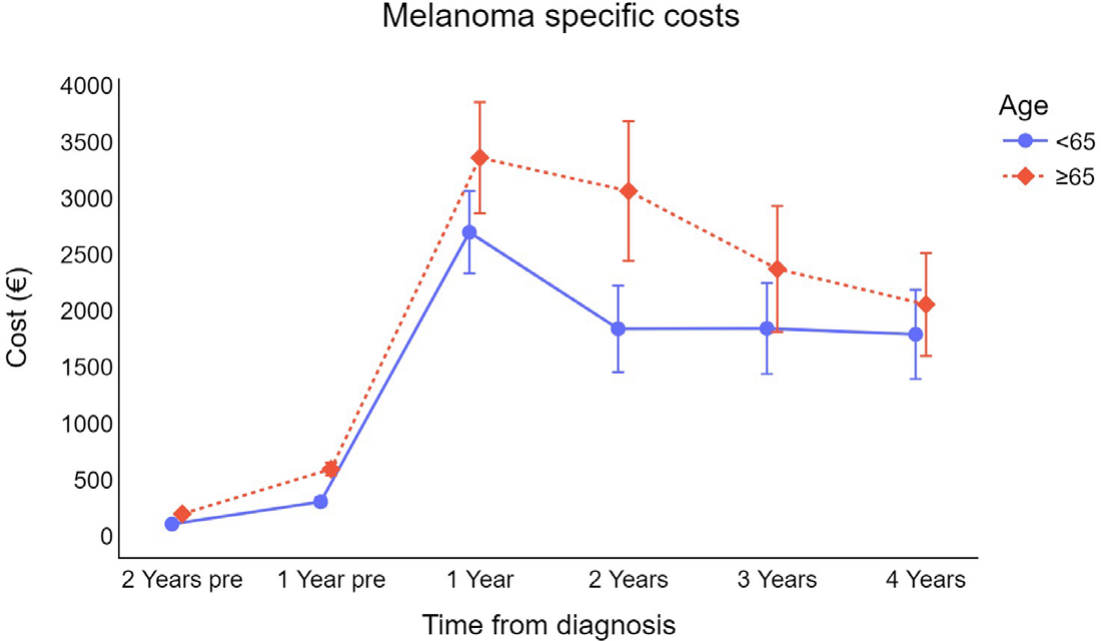
Trend for cutaneous melanoma survival-weighted mean cost per patient (in €) by age (error bars represent 95% CI for the mean). €, euros; CI, confidence interval.

On average, men incur higher direct melanoma costs than women (Fig. 1), and, as expected, the costs for older subjects (≥65 years) are generally higher than those for younger patients (<65 years) (Fig. 2).

**Fig. 3:**
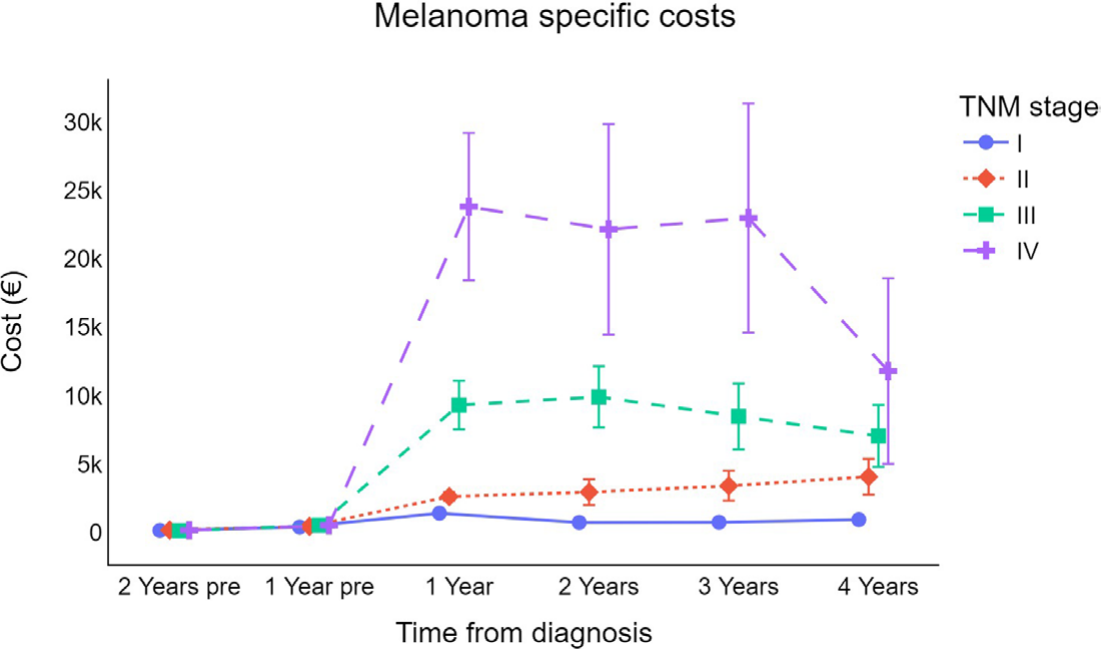
Trend for cutaneous melanoma survival-weighted mean cost per patient (in €) by TNM stage at diagnosis (error bars represent 95% CI for the mean). €, euros; CI, confidence interval; TNM, tumor-node-metastasis.

Fig. 3 depicts the cost curves for each TNM stage. Statistically, the survival-weighted mean expense per patient differs between stages I, II, III, and IV patients at 1 year (in sequence: €1405, 95% CI: 1339–1472; €2620, 95% CI: 2306–2933; €9313, 95% CI: 7535–11 090; €23 809, 95% CI: 18 418–29 199) and at 2 years following a CMM diagnosis (€728, 95% CI: 626–829; €2943, 95% CI: 1994–3892; €9907, 95% CI: 7676–12 139; €22 149, 95% CI: 14 451–29 847). In contrast to the other subgroups, the costs of stage II melanoma cases increase year after year.

**Fig. 4:**
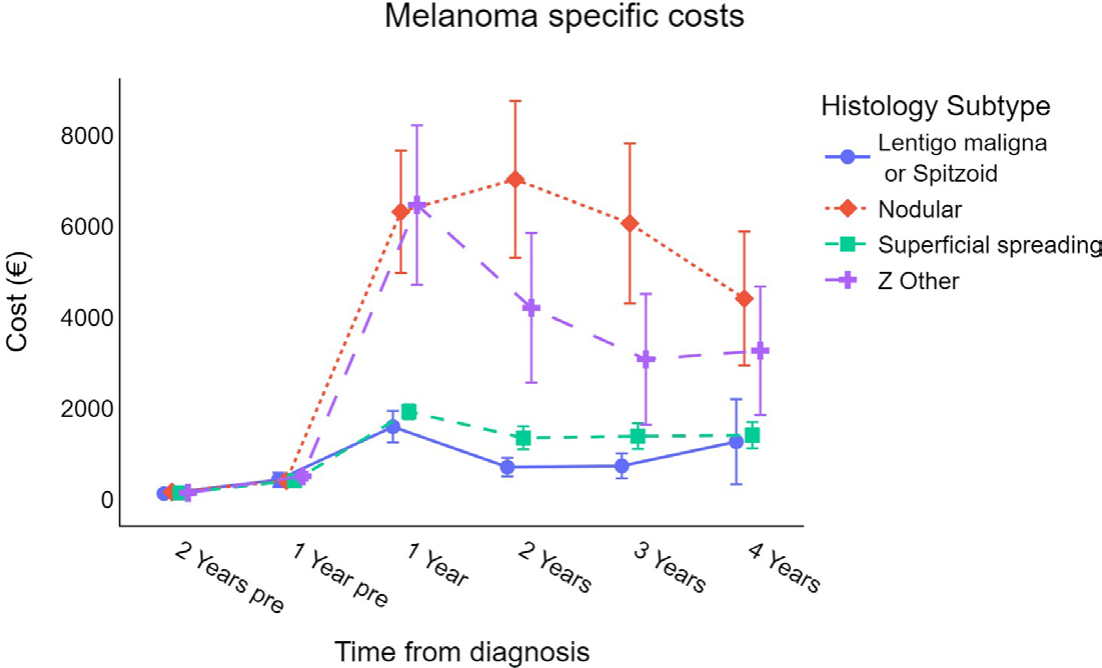
Trend for cutaneous melanoma survival-weighted mean cost per patient (in €) by histology subtype (error bars represent 95% CI for the mean). €, euros; CI, confidence interval.

Analyzing the differences in costs by histological subtype (Fig. 4), nodular and malignant melanomas not otherwise specified are associated with higher costs than superficial spreading, lentigo maligna, spitzoid, and other cell-differentiated melanomas.

**Fig. 5:**
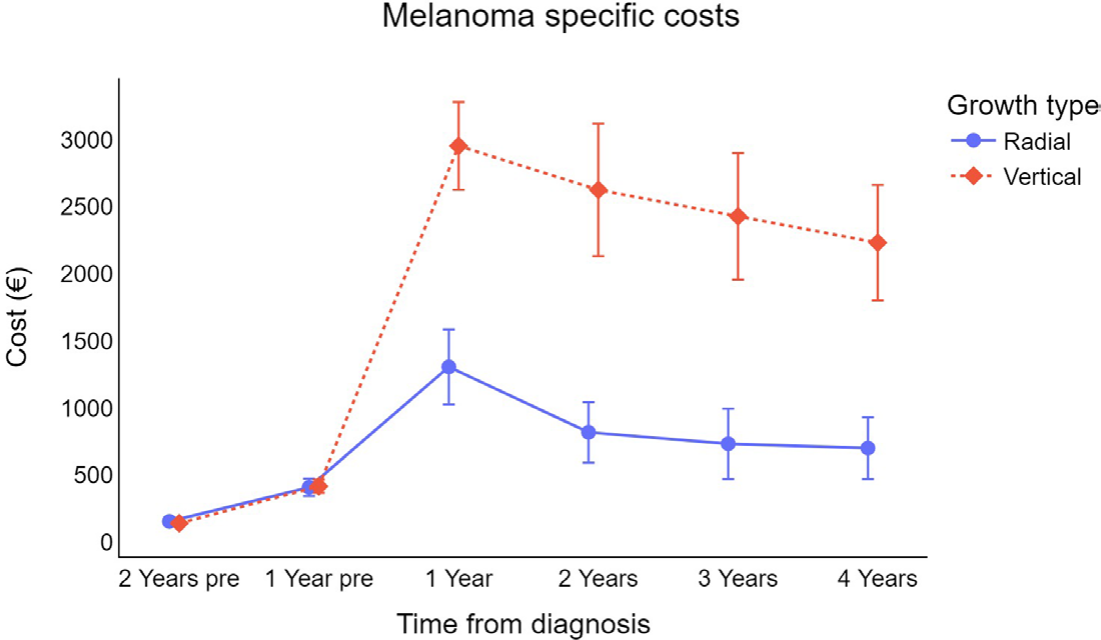
Trend for cutaneous melanoma-specific survival-weighted cost per patient (in €) by growth type. €, euros.

Concerning the difference in expenditure by growth type (Fig. 5), the direct costs of vertical growth melanoma are about three times higher than those of radially growing melanoma during the first, second, third, and fourth years following diagnosis.

**Fig. 6:**
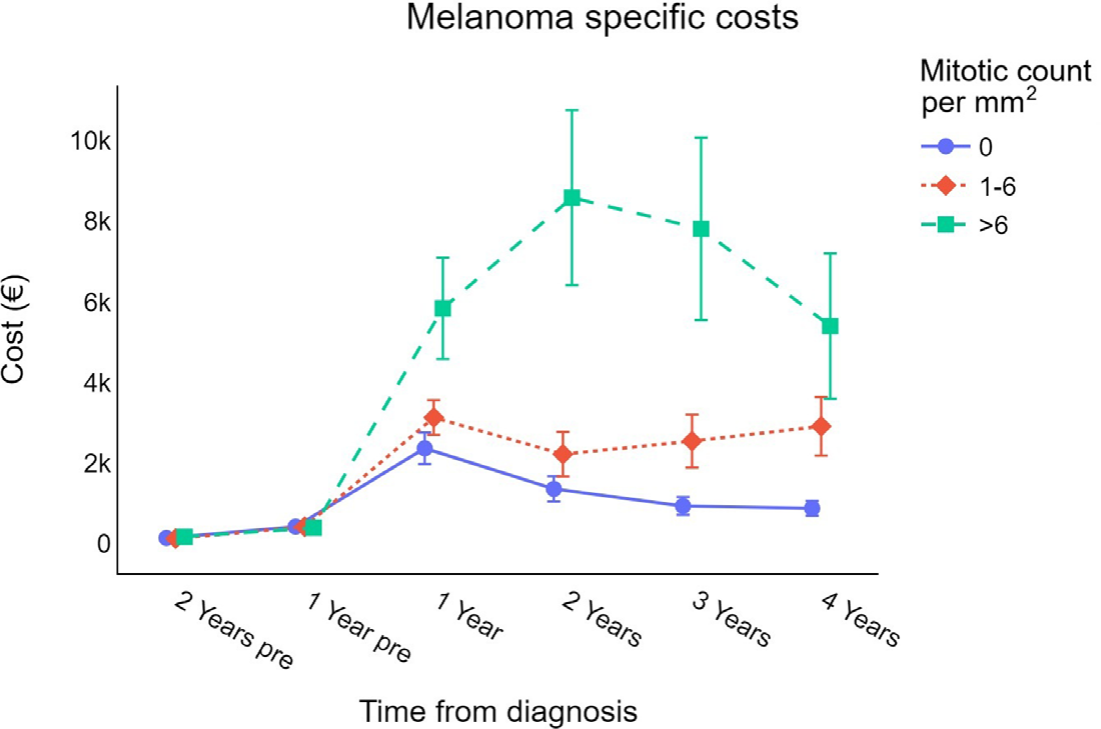
Trend for cutaneous melanoma-specific survival-weighted cost per patient (in €) by mitotic count per mm^3^. €, euros.

Cost differences by mitotic count are shown in Fig. 6; healthcare expenditure increases with the number of mitoses.

**Fig. 7:**
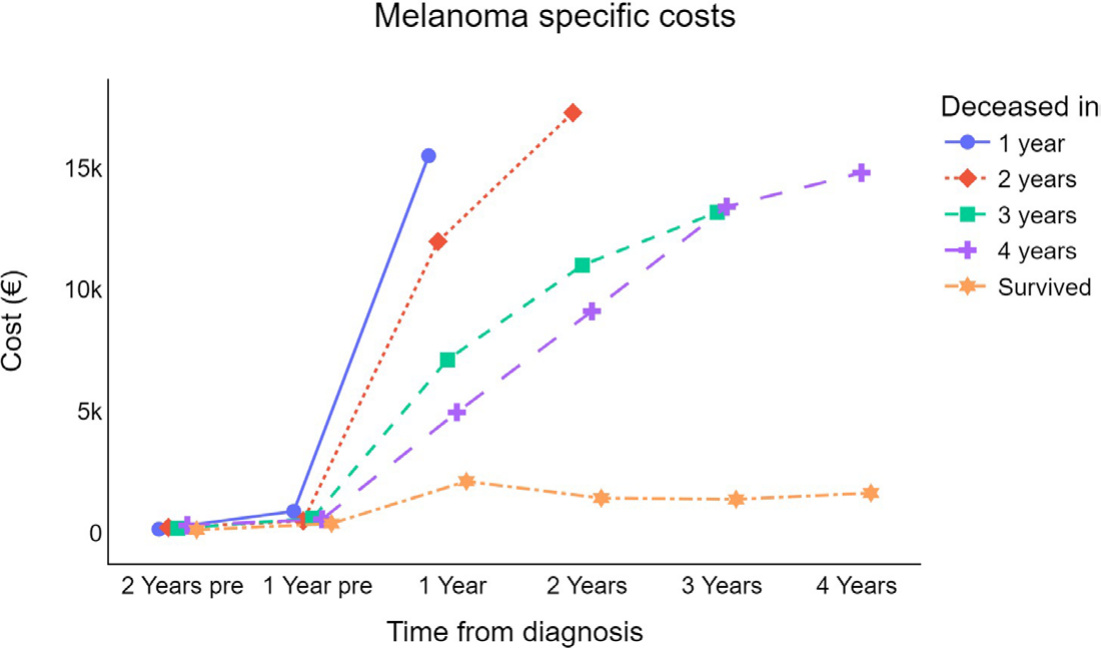
Trend for cutaneous melanoma-specific survival-weighted mean cost per patient (in €) by survival. €, euros.

Fig. 7 illustrates the costs influenced by survival. A rapid increase in costs is observed until the time of death. The peak cost per patient amounts to €15 494, €17 264, €13 175, and €14 797 when the terminal phases of care occur 1, 2, 3, and 4 years after diagnosis, respectively.

## Discussion

This study investigated the direct healthcare costs of patients with melanoma and found that these are higher for males and elderly patients, varying by phase of care and stage at diagnosis, with the highest costs incurred by patients diagnosed with late-stage disease and receiving terminal care. In terms of histological subtypes, nodular melanoma, and malignant melanoma not otherwise specified incurred the highest expense. In addition, direct costs increased with the mitotic count and vertical growth of melanoma lesions.

In terms of gender disparities in CMM-related costs, this study found that males had higher healthcare costs than females, which is consistent with previous research [14]. These data may be explained by a female-specific survival advantage that was previously demonstrated across all stages of the disease [21]. Men appeared to have a higher risk of distant metastasis and death from malignant melanoma [22], even after adjusting for other risk factors such as age, primary tumor site, clinicopathological type, and Breslow thickness [23]. One explanation for this phenomenon could be that men tend to have a lower propensity to prevent melanoma lesions by self-inspecting their skin and visiting healthcare providers. This may contribute to late detection [24].

In terms of the effect of age on the direct costs of melanoma healthcare, this study found that older patients incurred greater costs than those under 65 years. As can be expected, older patients tend to have a more advanced stage at diagnosis [25–27] and a higher prevalence of comorbidities [28,29], which can explain this difference in direct costs because these factors are known indicators of higher healthcare resource consumption [29].

As anticipated, the TNM stage at diagnosis had an impact on the direct healthcare costs of melanoma, with later-stage disease being associated with higher healthcare expenditure because of its strong association with survival and prognosis [30]. Specifically, the analyses of different cost trends by TNM stages reveal that TNM stage I melanoma expenditure reaches its peak 1 year after diagnosis and then returns to prediagnosis levels beginning from the second year after diagnosis. In contrast, stage II melanoma costs continue to increase gradually during the years after diagnosis but are still lower than those of the more advanced stages. The direct costs of stage III and IV melanoma are considerably higher, particularly in the first, second, and third years after diagnosis, with stage IV expenditures approximately double those of stage III. Other researchers have analyzed CMM direct healthcare costs by TNM stage at diagnosis, but results are not always comparable because of the heterogeneity of healthcare systems and costing methodologies across studies [5]. A previous study based on the Swedish cancer registry examined the total expenditure associated with all healthcare contacts, including inpatient, outpatient, and primary care, for patients with CMM diagnosed between 2005 and 2012 in the County of Östergötlandand. This study reported that CMM-related costs per patient-year ranged from €2670 (stage I) to €29 291 (stage IV) for the first year after diagnosis; thereafter, the mean healthcare costs decreased over time but remained significantly higher than in the general population for all clinical stages during the follow-up period [31]. Compared with this study, the direct healthcare costs reported in the Swedish research study are higher from stage I (€2670 vs. €1406) up to stage IV (€29 291 vs. €23 809). One plausible explanation is that in our region, both biopsies and wide local excisions on patients with melanoma can be offered in an ambulatory setting, thus eliminating hospitalization costs.

Nodular melanoma and malignant melanoma not otherwise specified incurred the highest costs compared with other histological subtypes. This is likely because of their greater invasiveness; in particular, nodular melanoma is known to have rapid growth [32,33], a higher capacity for metastasis, and a poor prognosis [34], all of which contribute to higher consumption of healthcare resources.

Regarding growth pattern, vertical growth melanomas are almost three times more expensive than radial growth melanomas, and this difference persists during the entire 4-year period following diagnosis. This result, consistent with that of a previous study [14], can be explained by the higher metastatic potential of vertical growth melanomas [35], which may prompt physicians to opt for more aggressive treatments, and by the greater intrinsic invasiveness of vertical growth, melanomas compared with horizontal growth neoplasms, which are biologically indolent (in the absence of regression) and lack the ability to metastasize [36].

Similarly, direct healthcare costs increase with the mitotic count; specifically, the difference in direct costs between melanomas with no mitosis and those with 1–6 mitoses per mm^2^ was statistically significant in the third and second years after diagnosis, whereas melanomas with more than six mitoses resulted in higher costs starting from the first year after diagnosis. This result demonstrates that melanomas with a higher mitotic count are more likely to be invasive [37], resulting in an increase in healthcare expenditure. Although the mitotic index is not included in the eighth edition of the AJCC TNM system, suggesting that it is optional to report this variable for prognostic purposes, this study confirms its crucial role in predicting survival and costs [38].

Finally, the survival-weighted mean cost per patient trend revealed a rapid increase in costs until the time of death. This result is in line with previous studies that identified an increase in the use of resources and costs of care in the end-of-life phase [39,40]. In fact, in the final stages of life, the clinical and assistance demands are because of the necessity for palliative care, hospice services, or hospitalization, which are acknowledged as major contributors to the increased economic end-of-life burden [41,42].

### Strengths and limitations

This research is based on a population-based cancer registry, which provides sample data with more external validity and statistical power than center-specific studies. While being based on real-world data obtained from hospital and administrative data flows, indirect costs, outpatient drugs, and medical device-related expenditures are not included.

## Conclusions

This study demonstrated that direct healthcare costs for patients with melanoma are associated with sociodemographic characteristics, such as male gender and older age, and anatomopathological factors, such as TNM stage, mitotic count, and growth pattern, with vertical growth melanoma incurring the highest costs. In terms of histological subtypes, the most expensive were nodular melanoma and malignant melanoma not otherwise specified. In addition, the heaviest monetary burden is in the first year after CMM diagnosis, as well as in the terminal phases of care for end-stage diseases. Given the rising incidence of melanoma, the analysis of real-world direct costs for a population-based cohort of patients is essential for informing decision-makers on how to better allocate healthcare resources. Moreover, the high cost of diagnosing advanced-stage CMM underscores the importance of promoting primary prevention and early detection.

## Acknowledgements

This research has received ‘Current Research’ funds from the Italian Ministry of Health to cover publication costs.

Conceptualization: A.B. and M.R. Methodology, software, formal analysis, and visualization: C.C. Investigation: C.C. and P.D.F. Data curation: C.C. and M.Z. Writing – original draft preparation: C.M.F, A.S., and G.G. Writing – review and editing: A.B. and S.M. Supervision: A.B., A.V., S.T., and C.R.R. Project administration: A.B. All authors have read and agreed to the published version of the manuscript.

The study adheres to the Declaration of Helsinki and Resolution No. 9/2016 of the Italian Personal Data Protection Authority, with the latter also confirming the permissibility of processing personal data for medical, biomedical, and epidemiological research, as well as the permissibility of using data concerning the status of people’s health in aggregate form in scientific studies. To protect privacy and anonymity, the Veneto Regional Authority removes all direct identifiers and replaces them with a code number in all datasets, while retaining the ability to link data from different administrative databases, but not going back up to the identification of the patient. In this case, according to Resolution No. 9/2016 of the Italian Personal Data Protection Authority, written consent from patients is not required.

Ethical approval for the study was obtained from the Veneto Oncological Institute’s Ethics Committee (No. 52/2016).

The data supporting this study’s findings are held by the Veneto Epidemiological Registry and were used under license for this work. However, they are not available to the general public. These data are nonetheless available from Manuel Zorzi upon reasonable request and subject to authorization from the Veneto Epidemiological Registry (Veneto Regional Authority)

### Conflicts of interest

There are no conflicts of interest.
